# Quasi-Bound States in the Continuum-Enabled Wideband Terahertz Molecular Fingerprint Sensing Using Graphene Metasurfaces

**DOI:** 10.3390/nano15151178

**Published:** 2025-07-30

**Authors:** Jing Zhao, Jiaxian Wang

**Affiliations:** 1 The Higher Educational Key Laboratory for Flexible Manufacturing Equipment Integration of Fujian Province, Xiamen Institute of Technology, Xiamen 361021, China; zhaojing2018@stu.xjtu.edu.cn; 2 Key Laboratory of Physical Electronics and Devices of Ministry of Education, School of Electronic Science and Engineering, Xi’an Jiaotong University, Xi’an 710049, China; 3 School of Artificial Intelligence, Xiamen Institute of Technology, Xiamen 361021, China

**Keywords:** terahertz sensing, graphene metasurface, quasi-bound states in the continuum, molecular fingerprint recognition

## Abstract

The unique molecular fingerprint spectral characteristics in the terahertz (THz) band provide distinct advantages for non-destructive and rapid biomolecular detection. However, conventional THz metasurface biosensors still face significant challenges in achieving highly sensitive and precise detection. This study proposes a sensing platform based on quasi-bound states in the continuum (Quasi-BIC), which enhances molecular fingerprint recognition through resonance amplification. We designed a symmetric graphene double-split square ring metasurface structure. By modulating the Fermi level of graphene, this system generated continuously tunable Quasi-BIC resonance peaks across a broad THz spectral range, achieving precise spectral overlap with the characteristic absorption lines of lactose (1.19 THz and 1.37 THz) and tyrosine (0.958 THz). The results demonstrated a remarkable 763-fold enhancement in absorption peak intensity through envelope analysis for analytes with 0.1 μm thickness, compared to conventional bare substrate detection. This terahertz BIC metasurface sensor demonstrates high detection sensitivity, holding significant application value in fields such as biomedical diagnosis, food safety, and pharmaceutical testing.

## 1. Introduction

Terahertz (THz) radiation (0.1–10 THz) enables the unique identification of organic biomolecules through their characteristic spectral fingerprints [1], making this technology indispensable for medical diagnostics [2,3,4], food safety monitoring [2], environmental sensing [5], industrial process control [6], and biomedical studies [7,8]. A critical challenge stems from the inherent scale disparity between THz wavelengths (30 μm–3 mm) and nanoscale biomolecular dimensions, which severely limits light–matter interaction efficiency. Traditional THz absorption spectroscopy using pelletized solid samples typically demands hundreds of milligrams to gram-level quantities [9,10,11], necessitating advanced field-enhancing architectures to detect trace analytes. Metasurfaces—precisely engineered subwavelength structures—overcome this limitation by generating localized electromagnetic field enhancements. These nanostructures enable the ultrasensitive detection of both nanoscale biochemical specimens and low-concentration gaseous compounds [12,13,14,15], bridging the scale gap between THz waves and molecular targets.

Bound states in the continuum (BICs) manifest as localized electromagnetic states coexisting within radiative continua while exhibiting suppressed energy leakage [16,17]. These non-radiative modes feature theoretically infinite quality factors (Q) and near-zero optical losses, positioning them as transformative tools for advanced photonic systems including ultra-sensitive biosensors, low-threshold lasers, and nonlinear optical devices. Quasi-BICs bridge theoretical BIC concepts with practical applications by introducing controlled symmetry-breaking perturbations—such as geometric modifications or material heterogeneities—to transform ideal BICs into experimentally realizable resonators with ultrahigh yet finite Q factors and negligible radiative dissipation [17,18,19,20].

Currently, most high-sensitivity sensors are primarily refractive index sensors, where a single-frequency resonance peak shifts according to the refractive index changes of trace analytes [17,21,22]. Recently, researchers have proposed parameter-multiplexed sensors capable of generating a series of resonance peaks to enhance wave–matter interactions [23,24,25,26,27]. The amplitudes of these resonance peaks vary with the absorption spectrum of the analyte, and their envelope matches the characteristic absorption spectral profile of the analyte—yet with significantly higher intensity than the unenhanced absorption spectrum. The multiplexed sensing scheme enables the significant enhancement of terahertz molecular fingerprint signals, offering promising potential for trace substance detection.

In 2019, Leitis et al. pioneered a quasi-BIC-driven all-dielectric metasurface achieving ultrahigh quality factors. Their angle-multiplexed design spanned a broadband mid-infrared spectral range, demonstrating a 50-fold amplification of absorption spectral amplitudes compared to conventional measurements through resonance envelope superposition [28]. Later that year, the team enhanced this platform using geometric multiplexing, attaining 60-fold spectral signal enhancement [29]. Zhu et al. advanced the field in 2020 by applying angle-multiplexed dielectric grating structures to amplify the terahertz absorption signatures of lactose and 2,4-DNT, achieving consistent 20-fold enhancement factors [30]. Subsequent developments saw Chang et al. (2022) integrate graphene-tuned C-shaped split-ring resonators with broadband micro-nano photonic sensors, enabling glucose fingerprint detection across a 1.5 THz spectral window through surface-sensitive resonance engineering [31]. The most recent innovation by Chen et al. (2023) introduced a frequency-selective plasmonic metasensor array using cross-shaped geometric multiplexing. This platform delivered targeted enhancements of 6.8-fold for D-carnitine and 7.3-fold for L-carnitine within the 0.95–2.0 THz band, establishing a new paradigm for chiral molecular discrimination [32].

Conventional multi-pixel geometric multiplexing demands the fabrication of complex micro/nano-antenna arrays, while angle-multiplexed metasurfaces rely on precision angular alignment—both approaches incur prohibitive system complexity and manufacturing costs. Furthermore, the material composition and structural parameters of traditional metallic/dielectric metasurfaces remain frozen post-fabrication [4,33], fundamentally restricting their spectral adaptability. These limitations collectively impede broadband resonance tunability in terahertz sensing systems, representing a critical technological bottleneck.

Graphene, a monolayer carbon allotrope, demonstrates gate-tunable conductivity and exceptional chip-integration compatibility [34]. Its metasurface configurations enable real-time performance reconfiguration via field-effect modulation (voltage bias or optical pumping) [35], dynamically optimizing sensing parameters for diverse detection scenarios. Dudek et al. (2021) demonstrated a graphene-based tunable hyperbolic microcavity with mid-infrared optical modulation capabilities [36]. Rahad et al. (2024) developed a graphene-metamaterial-based tunable broadband polarization-insensitive absorber for terahertz antenna applications [37]. Crucially, the atomic thickness of graphene resonators achieves analyte conformality unmatched by bulk materials [38,39,40], maximizing the surface coverage of trace molecules and amplifying light–matter interaction cross-sections.

This study proposes a graphene-metasurface-based terahertz broadband molecular fingerprint sensor. The sensor features a symmetric double-split square ring graphene structure capable of generating a series of quasi-BIC resonance peaks through Fermi level modulation. Refractive index sensing characterization demonstrates a sensitivity of 427 GHz/RIU with an outstanding figure of merit (FOM) reaching 15.2. The sensor achieved enhanced molecular fingerprint spectral acquisition across a broad frequency range, enabling the precise detection and identification of trace lactose and tyrosine molecules with an impressive detection limit as low as 100 nm. Compared with conventional substrate detection methods, the proposed metasurface provides remarkable signal enhancement up to 763-fold, offering an innovative approach for high-sensitivity fingerprint spectral recognition.

## 2. Structural Design and Method

**Figure 1:**
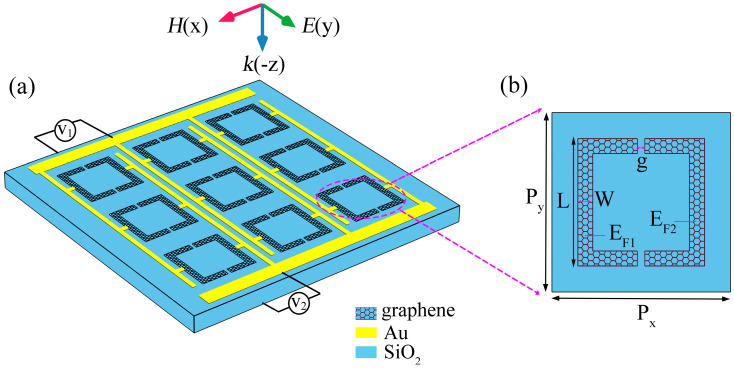
(**a**) Schematic illustration of the overall layout of the graphene symmetric double-split square ring metasurface; (**b**) schematic representation of the elemental structure.

The study is based on a terahertz sensing platform structure with a graphene metasurface, as shown in Figure 1a. The metasurface consists of periodically arranged symmetric graphene double-split square ring resonators deposited on a silicon dioxide (SiO_2_) substrate. In the terahertz frequency range, the refractive index of SiO_2_ is 1.95, and its imaginary part (loss) can be neglected; thus, it was not considered in the simulations. Figure 1b illustrates the unit cell structure of the sensor, with a period of P_x_ = P_y_ = 4.5 μm. The symmetric split square ring had a length of L = 3.4 μm, a width of W = 0.5 μm, and a split gap of g = 0.2 μm. The device employed gold (Au) as the electrode material with a thickness of 0.2 μm, and used a 10 μm-thick silicon dioxide (SiO_2_) layer as the spacer. To facilitate distinction, the Fermi levels of the left and right graphene structures in the unit cell were defined as E_F1_ and E_F2_, respectively. The Fermi level of graphene can be precisely controlled through electrical gating.

In the terahertz frequency range, planar graphene metasurfaces can excite quasi-BIC, whose confined electromagnetic fields significantly enhance light–matter interactions. The complex surface conductivity of graphene is determined using the Kubo formula [41,42]

(1) $$\begin{array}{l} \sigma_{g}=\sigma_{\mathrm{intra}}+\sigma_{\mathrm{int}er}\\ =\frac{-ie^{2}k_{B}T}{\pi \hbar^{2}(\omega -i/\tau )}\left[\frac{E_{F}}{k_{B}T}+2\ln\left(1+\exp(-\frac{E_{F}}{k_{B}T})\right)\right]+\frac{-ie^{2}}{2h}\ln\left[\frac{2|E_{F}|-\hbar (\omega -i/\tau )}{2|E_{F}|+\hbar (\omega -i/\tau )}\right] \end{array}$$

Here, *E_F_* is the graphene’s Fermi level, *ω* is the angular frequency, *e* is the electron charge, *T* is the temperature, ℏ is the reduced Planck constant, *h* is the Planck constant, *τ* is the carrier relaxation time, and $k_{B}$ is the Boltzmann constant. $\sigma_{\mathrm{int}ra}$ and $\sigma_{\mathrm{int}er}$ correspond to intraband and interband conductivities, respectively. The first and second equations describe the contributions from intraband electron transport and interband electron transport, respectively. In the THz range, the intraband conductivity dominates due to the Pauli blocking effect, which enhances the surface plasmon momentum and enables the propagation of surface plasmon waves in graphene. For simplification, we utilized the Drude model for the intraband contribution, expressed by the formula [34].

(2) $$\sigma_{g}=\frac{ie^{2}E_{F}}{\pi \hbar^{2}(\omega +i\tau^{-1})}$$

The carrier mobility is calculated using the formula $\mu =\tau {\left(ev_{F}\right)}^{2}/E_{F}$, where the Fermi velocity $v_{F}$ takes the typical value of 1 × 10^6^ m/s. For graphene with a Fermi level *E_F_* of 1 eV and a relaxation time τ of 10 ps, the calculated carrier mobility μ is approximately 1 × 10^5^ cm^2^·V^−1^·s^−1^. The value, though notably lower than the theoretical maximum [43] (by approximately one order of magnitude), remained practically viable as it matched the performance limits achievable with current fabrication techniques [44].

The relationship between the chemical potential (Fermi level $E_{F}$) of graphene and the gate voltage $V_{g}$ is given by the following equation [45,46]:

(3) $$E_{F}=\hbar v_{F}\sqrt{\pi \frac{C_{ox}V_{g}}{e}}$$

where $v_{F}$ is the Fermi velocity, $V_{g}$ is the gate voltage, and $C_{ox}$ is the oxide capacitance. Based on Equations (2) and (3), the conductivity of graphene can be actively modulated through gate voltage tuning, which directly influences its Fermi level.

In this study, we performed numerical simulations using the commercial finite element analysis software COMSOL Multiphysics 6.1, with the maximum mesh size deliberately set to be smaller than the critical feature dimensions of the structure to guarantee solution convergence. This study employed transition boundary conditions to implement the two-dimensional electromagnetic modeling of graphene, characterizing it as a nanoscale conductive boundary through the configuration of surface conductivity parameters. Compared with conventional three-dimensional bulk material discretization approaches, this boundary-condition-based modeling strategy significantly enhances numerical solving efficiency while maintaining computational accuracy. During the simulation, the incident field was a y-polarized plane wave that propagated down the *z* axis, with Floquet periodic boundary conditions imposed along the *x* and *y* axes.

## 3. Results Analysis and Discussion

A high-Q resonance peak implies that the electric field becomes highly localized, thereby significantly enhancing the interaction between terahertz waves and trace analytes on the metasurface. The Q-factor is evaluated as follows:

(4) $$Q=\frac{f_{0}}{\Delta f}$$

Here, *f* represents the resonance frequency, and Δ*f* is defined as the spectral width of the resonance peak at half of its maximum reflectivity.

**Figure 2:**
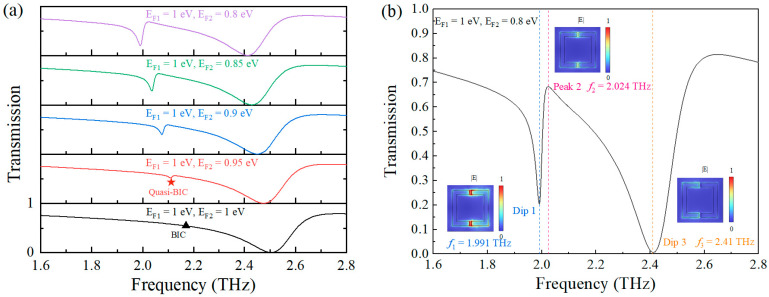
(**a**) Transmission spectra under different graphene Fermi levels for E_F2_ and E_F1_ = 1 eV; (**b**) Transmission spectra at E_F1_ = 1 eV, E_F2_ = 0.8 eV. The insets show the electric field energy density.

Figure 2a demonstrates Fermi-level-dependent transmission spectral evolution in the graphene metasurface. Initial symmetric configuration (E_F1_ = E_F2_ = 1 eV) exhibited a single broad resonance (1.6–2.8 THz range). Introducing Fermi level asymmetry (fixed E_F1_ = 1 eV, decreasing E_F2_) induced symmetry-broken quasi-BICs, manifesting as dual resonances: an ultra-narrow mode and broad background. The narrow resonance emerged through symmetry-breaking-induced radiation channel creation, enabling coupling between confined modes and free-space continuum—converting ideal BIC (black triangle, infinite Q) to high-Q quasi-BIC (red pentagram) via controlled radiative leakage. The ideal BIC regime (zero spectral linewidth) demonstrated complete decoupling from radiative states, characteristic of symmetry-protected BICs sustained by structural and excitation symmetry. Metasurface symmetry perturbation triggered a transition to quasi-BIC with finite radiation loss.

Progressive E_F2_ reduction reduced the transmission amplitude of Dip 1, achieving a transmission amplitude of 20% and a peak Q-factor of 66 at E_F2_ = 0.8 eV (EF_1_ = 1 eV). Figure 2b presents the corresponding transmission spectra and modal electric field distributions. Dip 1 exhibited strong field localization within the split-ring regions (inset), demonstrating enhanced field-matter overlap critical for sensing optimization. The behavior of Dip1 exhibited Fermi-level-dependent symmetry characteristics, which fundamentally explains its association with the quasi-bound state in the continuum (q-BIC) phenomenon [47]. Although Dip1 exhibited stronger Fermi level dependence than Dip3, this did not preclude its q-BIC nature. In fact, q-BIC modes can also demonstrate significant Fermi level dependence, particularly in two-dimensional materials such as graphene [48]. Therefore, the discussion of Dip1 as a q-BIC mode remains appropriate, as its behavior aligned with the fundamental properties of q-BIC phenomena.

**Figure 3:**
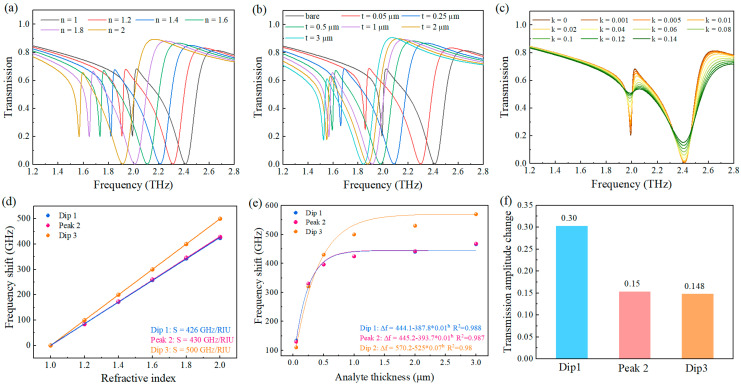
Transmission spectra for different (**a**) refractive indices, (**b**) thicknesses and (**c**) k values of the analyte; (**d**) frequency shift (Δ*f*) as a function of the refractive index; (**e**) relationship between the frequency shift and thickness; (**f**) amplitude changes for dip 1, peak 2, and dip 3. The graphene Fermi level is fixed at E_F1_ = 1 eV, E_F2_ = 0.8 eV.

We systematically investigated the influence of the analyte’s external environment on the resonance characteristics. In our simulations, the refractive index range was set from 1.0 to 2.0 to encompass typical biological molecules whose refractive indices generally fall within this range. The graphene metasurface was modeled in direct contact with a 1 μm thick analyte layer. The sensitivity of the sensor was defined as S=Δf/Δn (GHz/RIU), where Δf is the change in the resonant peak frequency, Δn is the refractive index change. FOM was used to evaluate the performance of the sensor, defined as FOM=S/FWHM(full-width-at-half-maximum). As shown in Figure 3a, dip 1, peak 2, and dip 3 all exhibited significant redshift phenomena, attributed to the dynamic capacitance changes induced by the analyte [49]. Figure 3d establishes a linear correlation between resonance frequency shifts and environmental refractive index changes. Linear regression analysis quantified the sensing performance across three characteristic modes: dip 1 (426 GHz/RIU), peak 2 (430 GHz/RIU), and dip 3 (500 GHz/RIU). The metasurface exhibited mode-dependent spectral linewidths with FWHM values of 30 GHz, 230 GHz, and 200 GHz respectively, translating to figure-of-merit (FOM) parameters of 14.2, 1.87, and 2.5. Thus, Dip 1 demonstrated a symmetry-protected transition from BIC to q-BIC, exhibiting a high Q-factor and narrow linewidth. In contrast, although Dip 3 displayed greater frequency shifts, it possessed a lower Q-factor, broader linewidth, and reduced FOM. For fingerprint spectrum sensing applications requiring high sensitivity and resolution to detect minute frequency variations, Dip 1′s characteristics rendered it more suitable for this field.

Figure 3b demonstrates analyte-thickness-dependent transmission spectral evolution. The thickness–resonance correlation plotted in Figure 3e revealed a characteristic redshift progression: resonance frequencies shifted nonlinearly with decaying rates as thickness increased, achieving saturation beyond 1 μm. This saturation threshold confirmed preserved sensor sensitivity even for submicron analyte layers.

We systematically examined the correlation between the extinction coefficient (k) and amplitude transmittance. As illustrated in Figure 3c, variations in k values from 0 to 0.14 produced distinct amplitude transmission spectra, where dip 1 showed particularly pronounced sensitivity to k-value modifications. This enhanced responsiveness significantly boosted the quasi-BIC resonance’s coupling efficiency with molecular vibrations, establishing an effective approach for high-precision material fingerprint detection. The comparative analysis of amplitude fluctuations in Figure 3f further confirmed that quasi-BIC resonance exhibited exceptional sensitivity to light–matter interactions, manifesting concurrent modifications in both amplitude characteristics and spectral positioning. Such dual-parameter responsiveness creates novel opportunities for developing advanced multimodal biosensing platforms with superior detection capabilities.

**Figure 4:**
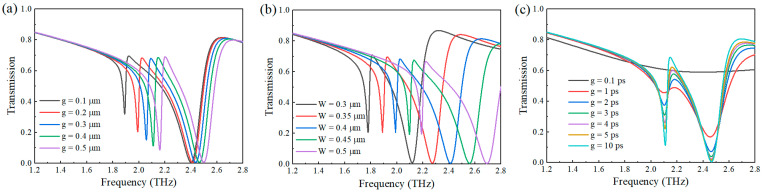
Dependence of terahertz transmittance on (**a**) split-ring resonator gap width, (**b**) metallic arm width, and (**c**) carrier relaxation time.

First, as shown in Figure 4a, we analyzed the effect of the split-ring gap dimension on the resonant properties. Through meticulous parametric studies, we observed that as the gap size (g) progressively increased from 0.1 μm to 0.5 μm, the resonant frequency demonstrated a consistent blueshift trend accompanied by a significant enhancement in transmission amplitude. This phenomenon suggests improved coupling efficiency between incident electromagnetic waves and the metamaterial structure at larger gap dimensions. After comprehensive consideration of the practical absorption losses, we determined the optimal gap size to be 0.2 μm, which achieved an optimal balance between resonant frequency position and transmission efficiency. Subsequently, we conducted detailed investigations on the impact of the split-ring width (W) on transmission properties (Figure 4b). The results revealed that increasing the width parameter from 0.3 μm to 0.5 μm induced a substantial blueshift in resonant frequency, with the shift magnitude being significantly more pronounced than that caused by gap variations. Notably, unlike the case with gap parameter changes, the transmission amplitude remained remarkably stable throughout the entire width variation range, showing less than 5% fluctuation. This distinct behavior clearly demonstrates that the split-ring width serves as the dominant geometric parameter for precise resonant frequency tuning while maintaining stable transmission amplitude characteristics. These systematic studies elucidate the correlation mechanism between the optical response of metamaterials and geometric parameters, providing important guidelines for device optimization and performance regulation in practical applications. Figure 4c demonstrates the relationship between transmittance and graphene carrier relaxation time. As shown, the amplitudes of Dip1 and Dip3 gradually decrease with increasing relaxation time. Notably, when the relaxation time exceeded 1 ps, Dip1 maintained a discernible resonance peak suitable for sensing applications. This observation confirms the robustness of our findings across a broad temporal scale, indicating the universal applicability of our conclusions.

**Figure 5:**
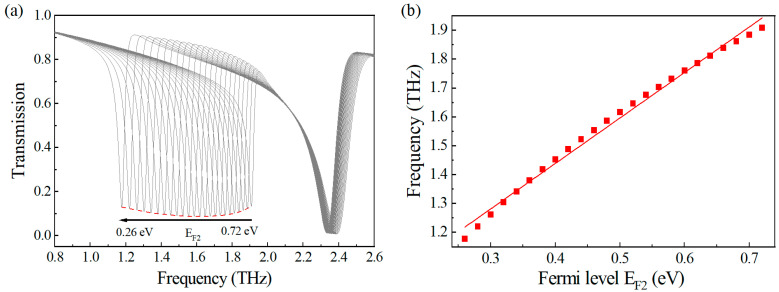
(**a**) The variation in the quasi-BIC resonance when E_F2_ decreases from 0.72 eV to 0.26 eV with a step size of 0.02 eV; (**b**) the relationship between the resonance frequency and the graphene’s Fermi level. E_F1_ is fixed at 1.0 eV.

To comprehensively investigate the spectral tuning characteristics of the quasi-bound states in the continuum (quasi-BIC) resonances, we performed systematic broadband spectral measurements under controlled Fermi level conditions. As clearly demonstrated in Figure 5a, while maintaining E_F1_ constant at 1.0 eV, we observed a continuous redshift of the resonant peak when E_F2_ was precisely tuned from 0.72 eV to 0.26 eV with fine increments of 0.02 eV. This phenomenon can be explained by the following formula: [50]: $\frac{1}{\lambda_{res}}\approx {\left(\frac{n\alpha_{0}E_{F}}{2\pi c\hbar cw}\right)}^{1/2}$, where n is the mode number, $\alpha_{0}$ is the fine-structure constant, ℏ is the reduced Planck constant, and c is the speed of light in vacuum. The red dashed curve represents the transmission envelope T_0_, which serves as a reference baseline for the resonance analysis.

The red dashed line in the Figure 5a represents the envelope of a series of resonant peaks. For enhanced visualization of the dynamic spectral evolution, we constructed Figure 5b to explicitly demonstrate the correlation between Fermi level modulation and resonance behavior. Our quantitative analysis revealed a remarkably linear dependence (R^2^ > 0.998) between the resonance frequency shift and graphene’s Fermi level within the operational frequency window of 1.2–1.9 THz. This linear tuning characteristic persisted across the entire parameter space we investigated, with a tuning sensitivity of approximately 1.5 THz/eV, as determined through linear regression analysis.

**Figure 6:**
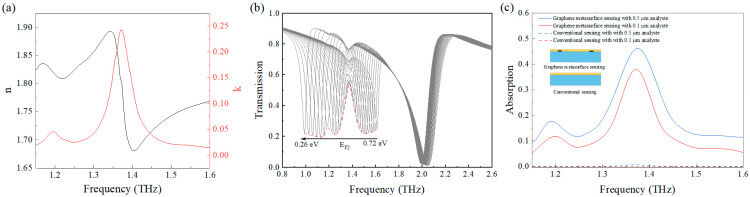
(**a**) Complex refractive index of the lactose molecules; (**b**) the transmission peak variations of lactose molecules coated on the metasurface as the graphene Fermi level changes; (**c**) the comparison between the proposed graphene metasurface sensing approach and traditional sensing methods.

To validate the effectiveness of the proposed metasurface in enhancing the terahertz absorption spectrum of trace analytes, α-lactose was used as the analyte for simulation validation. The dielectric permittivity of lactose was characterized using the Lorentz dispersion model [51].

(5) $$\varepsilon_{r}(\omega )=\varepsilon_{\infty }+\sum_{j=1}^{k}\frac{f_{j}\omega_{0j}^{2}}{\omega_{0j}^{2}-\omega^{2}-j\gamma_{j}\omega }$$

where *k* is the number of oscillators with angular resonance frequency and $\omega_{0j}$. $\gamma_{j}$ and $f_{j}$ are the damping constant and the oscillator strength of each absorption oscillation. $\varepsilon_{\infty }$ denotes the off-resonance background permittivity. The relationship between the dielectric permittivity and the refractive index is given by the following formula:

(6) $$\tilde{n}=n+ik=\sqrt{{\tilde{\varepsilon }}_{r}}$$

where *n* is the real part of the refractive index, representing the phase velocity of light in the material, *k* is the extinction coefficient, representing the absorption of light. Figure 6a shows the refractive index of lactose molecules. The black solid line represents the real part of the complex refractive index (n), while the red solid line corresponds to the imaginary part (k). It could be observed that within the range of 1.15–1.6 THz, lactose molecules exhibited two strong absorption signals at 1.19 THz and 1.37 THz [52].

The dashed line in Figure 6c presents the terahertz absorption characteristics of α-lactose films deposited on silicon dioxide substrates. Spectral analysis revealed extremely weak absorption signals at the characteristic lactose resonance frequency of 1.37 THz, with absorption coefficients of merely 0.0005 for the 0.1 μm film and 0.007 for the 0.5 μm film. In practical measurements, such faint signals are easily overwhelmed by noise, making detection challenging. To overcome this limitation, we employed a specially designed metasurface platform that generates strongly localized electromagnetic fields through carefully engineered subwavelength resonators, significantly enhancing light–matter interactions with the analyte molecules. A 0.5 μm lactose film was uniformly prepared on the metasurface and measured with vertically incident terahertz waves. Figure 6b demonstrates the dynamic tunability of the system, where progressive adjustment of the graphene Fermi level (E_F2_) from 0.26 eV to 0.72 eV induced substantial variations in the metasurface’s transmission resonance amplitude. The red dashed line in Figure 6b represents the transmittance envelope T_1_. The absorption spectrum was calculated using the relation A = T_1_ − T_0_, where T_0_ denotes the reference transmittance of the uncoated metasurface. Remarkably, the absorption spectra of both the 0.1 μm (red solid line) and 0.5 μm (blue solid line) lactose films (Figure 6c) showed excellent agreement with the reference spectrum of pure lactose (Figure 6a), confirming the graphene metasurface sensor’s capability to accurately identify lactose molecules.

Compared to conventional sensing methods, the metasurface enhancement technique achieved 763-fold and 66-fold increases in absorption peak intensity for the 0.1 μm and 0.5 μm films, respectively. This remarkable improvement stems from multiple synergistic mechanisms: (1) The localized field enhancement effect of the metasurface structure substantially strengthens light–matter interactions; (2) the tunability of graphene’s Fermi level enables precise control of the resonance conditions. These characteristics make this sensor highly valuable for biomolecular detection applications.

**Figure 7:**
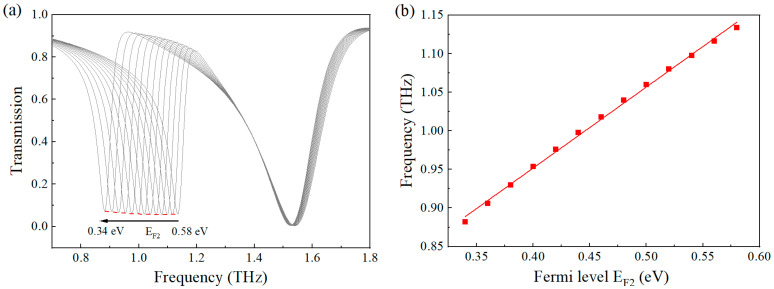
(**a**) The variation in the quasi-BIC peak when E_F2_ decreases from 0.58 eV to 0.34 eV with a step size of 0.02 eV; (**b**) the relationship between the resonance frequency and the graphene’s Fermi level. EF_1_ is fixed at 1.0 eV.

To further validate the universal detection capability of this graphene-based metasurface sensor, we systematically investigated its performance for tyrosine molecular detection. The metasurface structure was specifically optimized with the following geometric parameters: periodicity P_x_ = P_y_ = 10 μm, side length L = 8 μm and arm width W = 1 μm for the symmetric split-square resonator, and gap spacing g = 0.5 μm. Through precise electrostatic gating control, we tuned the graphene Fermi level within the range of 0.34–0.58 eV, resulting in a corresponding resonant frequency shift from 0.85 THz to 1.05 THz—completely covering the characteristic absorption band of tyrosine molecules. As shown in Figure 7a, the resonant frequency demonstrated a highly linear dependence (R^2^ = 0.996) on the graphene Fermi level, with a tuning sensitivity of 1.05 THz/eV as determined by linear regression analysis.

**Figure 8:**
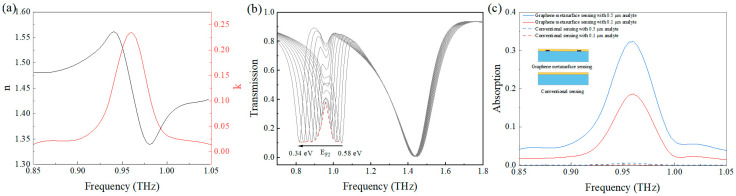
(**a**) Complex refractive index of the tyrosine molecules; (**b**) the transmission peak variations of lactose molecules coated on the metasurface as the graphene Fermi level changes; (**c**) the comparison between the proposed graphene metasurface sensing approach and traditional sensing methods.

The optical parameters of tyrosine are shown in Figure 8a. Within the frequency range of 0.85–1.05 THz, tyrosine molecules exhibit a strong absorption peak at 0.958 THz [53]. Similarly, a 0.5 μm tyrosine layer was deposited on the graphene metasurface under vertical terahertz wave incidence. As the E_F2_ increased from 0.34 eV to 0.58 eV, significant variations in the transmission resonance peak amplitude were observed. The derived absorption spectrum exhibited strong absorption at 0.958 THz, matching the reference absorption peak. Figure 8c demonstrates the thickness-dependent absorption intensity for the analyte layers of 0.1 and 0.5 μm, showing decreased but still prominent absorption even at the minimal 0.1 μm thickness. Compared to conventional sensing methods, the metasurface enhancement technique achieved 548-fold and 67-fold increases in absorption peak intensity for the 0.1 μm and 0.5 μm films, respectively. To demonstrate the superior performance of our graphene metasurface sensing platform for trace molecular fingerprint detection, we conducted a comprehensive performance comparison with state-of-the-art approaches in Table 1.

## 4. Conclusions

In conclusion, this study presents a quasi-bound state in the continuum (quasi-BIC)-enhanced terahertz trace molecular sensing platform, validated through theoretical modeling and numerical simulation. The sensor utilizes a periodic symmetric graphene double-split square ring metasurface, where controlled Fermi level modulation (0.26–0.72 eV) in one graphene layer while maintaining the counterpart at 1.0 eV enables tunable high-Q quasi-BIC resonance generation across the 1.2–1.9 THz spectral range. This architecture achieved a sensitivity of 427 GHz/RIU and a figure of merit (FOM) of 15.2. The result demonstrations revealed 763-fold and 548-fold enhancements in molecular vibrational signals for 0.1μm thick lactose and tyrosine analytes, respectively, through precise spectral matching with their characteristic absorption fingerprints. The platform establishes a new paradigm for ultra-sensitive trace biomolecular detection, with transformative potential in chemical/biological sensing applications. Although the proposed molecular detection system demonstrated excellent performance with solid thin-film samples, it still has certain limitations in practical applications. Currently, the system has only been validated using 0.1 μm thick solid lactose/tyrosine films and has not yet been tested in complex biological fluids such as serum or cell lysates, where background absorption interference may significantly affect detection results. To enhance its practical utility, future development should focus on two key improvements: (1) integrating microfluidic technologies to enable the precise handling of liquid samples and improve detection sensitivity by minimizing matrix effects, and (2) creating field-deployable solutions by incorporating portable devices with real-time monitoring capabilities to meet point-of-care testing requirements. This dual approach will address current limitations while expanding the system’s clinical and diagnostic potential.

## Figures and Tables

**Table 1:** Performance comparison of graphene-based sensors vs. conventional sensors.

Ref.	Unit Structure	Analyte	Multiplexing Method	Working Band	Enhance Factor
[30]	Dielectric metagrating	α-Lactose	Incident angle	THz	~9 times
[24]	Dielectric square nanodisks	Protein A/G	Incident angle	Mid infrared	~10 times
[32]	Metal crossed-slot	α-Lactose	Geometry	THz	~10 times
[31]	Graphene/metal C-shape	Glucose	Geometry/graphene Fermi level	THz	~5 times
[54]	Metal groove array	α-Lactose	Geometry	THz	~120 times
This work	Graphene spit ring	α-Lactose/tyrosine	Graphene Fermi level	THz	~763/~548 times

## Data Availability

The data that support the findings of this study are available from the corresponding author upon reasonable request.
